# Sodium Tanshinone IIA Sulfonate Attenuates Erectile Dysfunction in Rats with Hyperlipidemia

**DOI:** 10.1155/2020/7286958

**Published:** 2020-03-04

**Authors:** Liren Zhong, Wei Ding, Qingyu Zeng, Binglin He, Haibo Zhang, Li Wang, Junhong Fan, Shuhua He, Yuanyuan Zhang, Anyang Wei

**Affiliations:** ^1^Department of Urology, Nanfang Hospital, Southern Medical University, Guangzhou, Guangdong, China; ^2^Department of Urology, The First Affiliated Hospital of Guizhou University of Traditional Chinese Medicine, Guiyang, Guizhou, China; ^3^Wake Forest Institute of Regenerative Medicine University, Wake Forest School of Medicine, Winston-Salem, North Carolina, USA

## Abstract

Hyperlipidemia is considered one of the most important risk factors for erectile dysfunction (ED). To determine the effect of sodium tanshinone IIA sulfonate (STS) as an antioxidant agent on ED in high-fat diet- (HFD-) induced hyperlipidemia in rats and to investigate if STS administration could improve erectile function via hydrogen sulfide (H_2_S) production by inhibition of oxidative stress. Hyperlipidemia was induced in Sprague-Dawley rats by feeding HFD for 16 weeks. The rats were randomly divided into 3 groups: control, HFD, and HFD treated with STS (10 mg/kg/day for 12 weeks, intraperitoneal injection). Erectile function including intracavernosal pressure (ICP), H_2_S production, and antioxidant capacity was assessed. In addition, cavernosal smooth muscle cells (CSMC) isolated from SD rats were pretreated with STS in vitro and exposed to H_2_O_2_. Expressions of nuclear factor erythroid 2-related factor 2 (Nrf2)/heme oxygenase-1 (HO-1), activity of antioxidant enzymes, and H_2_S-generating enzymes within CSMC were examined. ICP was significantly decreased in HFD rats compared with control. In addition, decreased H_2_S production and expression of cystathionine *ɣ*-lyase (CSE) and cystathionine *β*-synthase (CBS) associated with increased oxidative stress were observed in the penile tissue of HFD rats. However, all these changes were reversed by 16 weeks after STS administration. STS also increased antioxidant defense as evidenced by increased expression of Nrf2/HO-1 in the penile tissue of HFD rats. In CSMC, pretreatment with STS attenuated the decreased expression of CSE and CBS and H_2_S production by H_2_O_2_. STS exerted similar protective antioxidative effect as shown in the in vivo hyperlipidemia model. The present study demonstrated the redox effect of STS treatment on ED via increased H_2_S production in HFD-induced hyperlipidemia rat model by increased antioxidant capacity via activation of the Nrf2/HO-1 pathway, which provides STS potential clinical application in the treatment of hyperlipidemia-related ED.

## 1. Introduction

Despite many causes of erectile dysfunction (ED), such as neurovascular injury, diabetes, vascular disease, drugs and substance abuse, and testosterone deficiency, hyperlipidemia due to high-fat diets (HFD) is considered one of the most important risk factors [1]. Prevalence of ED has increased linearly with the severity of hyperlipidemia [2]. Elevated levels of lipids in the blood is harmful, because lipids exposed to oxygen will go to the peroxidation process, which is a chain reaction that contributes to produced reactive oxygen species (ROS). Increased oxidative stress, resulting from elevated ROS production, appears to play an important role in hyperlipidemia-associated ED [3].

Hydrogen sulfide (H_2_S) was considered a toxic pollutant until 2001, when it was firstly recognized as a mediator of vasodilation [4]. H_2_S, the third most important biological gasotransmitter after nitric oxide (NO) and carbon monoxide (CO), is mainly synthesized endogenously from L-cysteine by two enzymes: cystathionine *β*-synthase (CBS) and cystathionine *ɣ*-lyase (CSE) in human penile tissue [5]. Recently, another enzyme, 3-mercaptopyruvate sulphurtransferase (3-MST), has been proposed to synthesize H_2_S [6]. Evidences suggest that H_2_S exerts important biophysiological effect on erectile dysfunction. A study in a nonhuman primate model showed that administration of sodium hydrosulfide hydrate (H_2_S donor) increases intracavernosal pressure (ICP) and penile length [7]. Such proerectile effect of H_2_S has also been observed in other animal model [8], whereas CSE or CBS inhibitors result in decrease in ICP and contraction of the corpus cavernosum (CC) [5], suggesting an indispensable role for CSE and CBS in the biophysiological effect of H_2_S. Recent study suggested that Western high-fat diet induces oxidative stress, which occurs alongside a reduction of CSE expression and H_2_S production in the vascular system [9]. However, limited information has reported on the endogenous H_2_S system in hyperlipidemia-associated ED.

Sodium tanshinone IIA sulfonate (STS) is a water-soluble derivative of tanshinone IIA, which is the lipophilic diterpene that is isolated from the root of Salvia miltiorrhiza (Danshen) plant. This plant is a traditional Chinese medicinal herb, which is effective in the treatment of various cardiovascular diseases (CVD), such as hyperlipidemia, ischemic stroke, and myocardial infarctions [10]. STS was found to increase antioxidant capacity by induction of nuclear factor erythroid 2-related factor 2 (Nrf2) and heme oxygenase-1 (HO-1) expression, which is involved in interference against oxidative stress [11].

Since H_2_S is an important regulator of erectile function and a proerectile molecule, examining how hyperlipidemia and subsequent oxidative stress affect its production and synthetic enzymes will examine whether or not H_2_S donors have the potential to be beneficial therapeutic agents for hyperlipidemia-associated ED. In addition, given the antioxidative effect of STS, we hypothesized that STS would have an ameliorating effect on ED in rats with hyperlipidemia through increasing H_2_S production by antioxidant actions of STS. Therefore, our current study will evaluate the involvement of STS's antioxidative effect in mediating H_2_S formation under hyperlipidemia or oxidative stress conditions, using a HFD-fed hyperlipidemia rat model in vivo and a hydrogen peroxide- (H_2_O_2_-) stimulated oxidative stress cavernosal smooth muscle cell (CSMC) model.

## 2. Materials and Methods

### 2.1. Animals and Experimental Design

All procedures were approved by the Animal Care and Use Committee of Southern Medical University (Guangzhou, China). The rats were maintained under a standard condition of temperature (22-25°C), a humidity of 60% ± 10%, and a 12-hour light/dark cycle. In all, a total of 36 male Sprague-Dawley rats weighing 250-300 g were randomized into 3 groups (12 in each group): normal control group (NC); high-fat diet group (HFD); and HFD treated with STS (10 mg/kg/day, intraperitoneal injection). To induce the hyperlipidemia models, the rats were fed with high-fat diet for 16 weeks continually. The ingredients in high-fat diet included 2% cholesterol, 10% lard, 10% egg yolk powder, and 79% standard rat chow (Zeigler Brothers, Gardner, PA, USA). The control rats received the basal diet at the same time.

### 2.2. Erectile Function Evaluation

After the 12-week treatment, erectile function was assessed using intracavernosal pressure (ICP) and mean arterial pressure (MAP) tests as previously reported [12]. Briefly, while under anesthesia, the bilateral cavernous nerves were exposed and a bipolar steel electrode was used for nerve stimulation. The corpora at the middle of penile shaft were cannulated with a 25-G needle containing heparin (100 U/ml) connected to a pressure transducer (Biopac Systems Inc., Goleta, California, USA) to record ICP. Stimulation parameters were 5 mA, 20 Hz, plus 0.2 ms, and duration of 60 s. The mean MAP was calculated after electrical stimulation. The erectile evaluation was measured as mean ICP, MAP, and the ratio of ICP and MAP. The mean ICP and MAP were recorded using AcqKnowledge®4.4 software (Biopac Systems Inc. Goleta, California, USA). After data recording, the blood samples and the shaft of the penis were harvested for analysis.

### 2.3. Cell Culture and Treatment

Primary CSMC were isolated from the corpus cavernosum of male Sprague-Dawley rats and identified as previously reported [13]. Isolated CSMC were cultured in DMEM containing 20% fetal bovine serum (Invitrogen, Carlsbad, CA, USA) at 37°C and 5% CO_2_. The purity and identity of CSMC were verified using a monoclonal antibody against calponin (1 : 100, Santa Cruz, USA). Cells from passages 3-4 were used in all experiments. To evaluate the protective effects of STS, the cells were pretreated with different doses of STS for 2 hours before H_2_O_2_ was added to the medium. The CSMC were then exposed to 100 *μ*M/ml H_2_O_2_ for 12 hours.

### 2.4. Cell Viability Assay

The cell viability of CSMC was determined by using a CCK8 assay kit (Dojindo, Japan) according to the manufacturer's instruction. Briefly, the cells (5,000 cells/well) were seeded in 96-well plates, cultured overnight, and treated with H_2_O_2_ (0-400 *μ*M/ml) for 6, 12, and 18 hours or STS (0-60 *μ*g/ml) for 48 hours. Subsequently, CCK8 solution (10 *μ*l) was added to each well and incubated for 2 hours. The absorbance at 450 nm was read in a microplate absorbance reader (Thermo Scientific, Waltham, MA). Cell viability was presented as percentage of normal control cells.

### 2.5. H_2_S Measurement

Levels of H_2_S in tissues or cells were measured as previously described [14]. The penile tissue or CSMC were homogenized in 2 ml of 100 mM potassium phosphate buffer (pH 7.4). Homogenates (430 *μ*l) were added in a reaction mixture (500 *μ*l) containing 2 mM pyridoxal 5′'-phosphate (20 *μ*l), 10 mM L-cysteine (20 *μ*l), and PBS (30 *μ*l). The reaction was performed at 37°C for 30 min in tightly sealed Eppendorf vials. Zinc acetate (1%, 250 *μ*l) was then injected to trap generated H_2_S followed by trichloroacetic acid (10%, 250 *μ*l) to precipitate protein and thus stop the reaction. Subsequently, N,N-dimethyl-p-phenylenediamine sulfate (NNDPD) (20 mM, 133 *μ*l) in 7.2 M HCl and FeCl_3_ (30 mM, 133 *μ*l) in 1.2 M HCl were added, and absorbance (670 nm) of aliquots of the resulting solution was determined. All samples were assayed in duplicate, and H_2_S concentration was calculated against a calibration curve of NaHS (3.125-250 *μ*M) and expressed in *μ*M/g.

Plasma was centrifuged at 4000 rpm for 4 min at 4°C. Supernatant (100 *μ*l) was added to a tube containing zinc acetate (1%, 250 *μ*l) and distilled water (400 *μ*l). The reaction mixture was incubated at 37°C for 30 min with NNDPD (20 mM, 133 *μ*l) in 7.2 M HCl and FeCl_3_ (30 mM, 133 *μ*l) in 1.2 M HCl. The reaction was stopped, and the samples were then centrifuged at 14,000 rpm for 10 min at 4°C. The absorbance (670 nm) of aliquot of the resulting supernatant was read. Method of measurement of concentration was the same as mentioned above, and the plasma H_2_S concentration was expressed in *μ*M.

### 2.6. WSP-1

To verify the results of extracellular H_2_S, intracellular H_2_S contents of the penile tissue or CSMC were visualized using the fluorescent probe WSP-1 (Cayman Chemical, Ann Arbor, MI, USA) as previously reported with some modifications [15]. Briefly, cells or paraffin section of tissues were washed with PBS for three times and incubated with 150 *μ*M WSP-1 at 37°C for 30 min in the dark. The fluorescence intensity was observed by Olympus laser scanning confocal microscopy and analyzed by ImageJ software (National Institutes of Health, Bethesda, MD, USA).

### 2.7. Immunofluorescence Analysis and Confocal Microscopy

For immunofluorescence analysis, fresh tissues were immersed in OCT (Leica Biosystems, Buffalo Grove, IL, USA), frozen in liquid nitrogen, and stored at -80°C. The midshaft of the penis was used to prepare 5 *μ*m sections, which were adhered to slides. Then, the tissue sections and CSMC grown on coverslips were fixed with 4% paraformaldehyde for 15 min, permeabilized in 0.2% Triton X-100 for 10 min, and blocked for 1 h with 1% BSA. Thereafter, cells were incubated with primary antibody overnight at 4°C, followed by double staining of secondary antibodies at 37°C for 1 hour. The information for antibodies is listed in Table 1. Nuclei were stained with DAPI (Abcam, Cambridge, MA, USA). Images were captured using Olympus laser scanning confocal microscopy.

### 2.8. Biochemical Assay

The CSMC and the penile tissue were homogenized in Tris-HCl buffer (pH = 7.4) and centrifuged at 14,000 rpm for 10 min at 4°C. The supernatants were collected for biochemical analysis. Malondialdehyde (MDA), glutathione (GSH), and superoxide dismutase (SOD) were measured using three commercial detection kits according to the manufacturer's instructions and previously described methods [16].

Other serum markers including triglycerides (TG), total cholesterol (TC), low-density lipoprotein cholesterol (LDL-C), and high-density lipoprotein cholesterol (HDL-C) levels were spectrophotometrically measured using commercial assay kits as described before [17].

### 2.9. Western Blotting

The expression of HO-1, Nrf2, CSE, CBS, and 3-MST was determined by Western blotting according to previously described methods [18]. The information for the primary antibodies is listed in Table 1. Secondary antibodies were purchased from Abbkine Inc. (catalog # A21010, # A21020, USA) and diluted at 1 : 30,000. Nuclear protein was isolated from the cells using a nuclear extraction kit following the manufacturer's instructions. Blots were analyzed with an enhanced chemiluminescence detection system.

### 2.10. Statistical Analysis

Statistical analysis was performed with the SPSS version 21.0 software for Windows. Results were recorded as mean ± standard error (SEM). Given the skewed distribution of data and the modest sample size, the nonparametric Kruskal–Wallis test and Mann–Whitney *U* test were used to assess statistical significance. *P* < 0.05 was considered statistically significant.

## 3. Results

### 3.1. Effect of STS on Body Weight and Serum Lipid Levels

**Figure 1:**
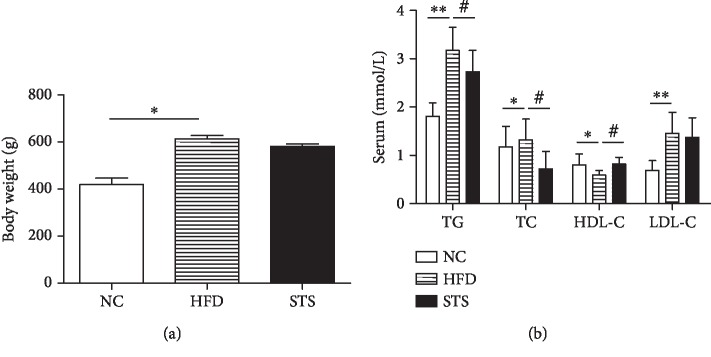
Metabolic variables. (a) Body weight after 16 weeks' feeding. (b) Serum lipid levels in each group. Each bar represents mean ± SEM; *N* = 12 rats per group. ^∗^*P* < 0.05 compared to the NC group; ^∗∗^*P* < 0.01 compared to the NC group; ^#^*P* < 0.05 compared with the HFD group.

The body weight of HFD rats was significantly higher than that of the NC control rats 16 weeks after feeding (*P* < 0.05, Figure 1(a)). The animals fed high-fat diet showed significantly higher levels of all the serum lipids levels except for HDL-C, which showed a reduction (*P* < 0.05, Figure 1(b)). The body weight gain observed in the rats treated with STS did not show significant changes (*P* > 0.05, Figure 1(a)). However, STS treatment significantly reduced the TG and TC levels and increased the HDL-C level (*P* < 0.05, Figure 1(b)).

### 3.2. Impact of STS on Impaired Erectile Function in HFD Rats

**Figure 2:**
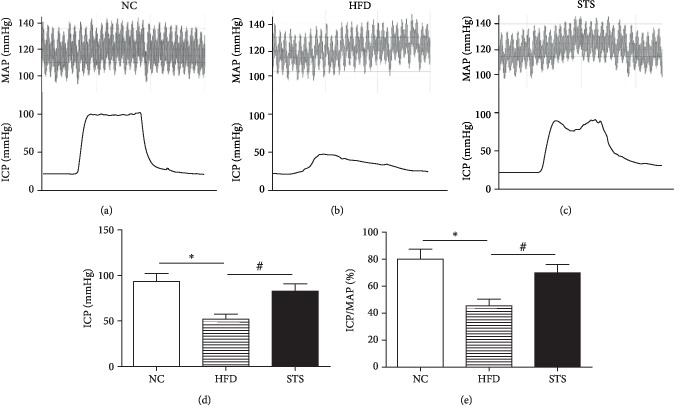
Erectile function assessed by ICP/MAP. (a–c) Representative tracings of ICP in response to electrostimulation of cavernous nerve and MAP for each group. The black bar denotes the electrical stimulation. The red curve above denotes the MAP during the electrostimulation. (d, e) Erectile function is presented as ICP and ICP/MAP. Each bar represents mean ± SEM; *N* = 12 rats per group. ^∗^*P* < 0.05 compared to the NC group; ^#^*P* < 0.05 compared with the HFD group.

Erectile function was significantly decreased in the HFD group, as evidenced by decreased average ICP and ICP/MAP ratio relative to NC rats (*P* < 0.05, Figure 2(a)). STS treatment significantly improved erectile function greater than 1.5-fold compared to that in rats in the HFD group (*P* < 0.05, Figure 2(b)).

### 3.3. H_2_S Level and Endogenous H_2_S Generation in HFD Rats

**Figure 3:**
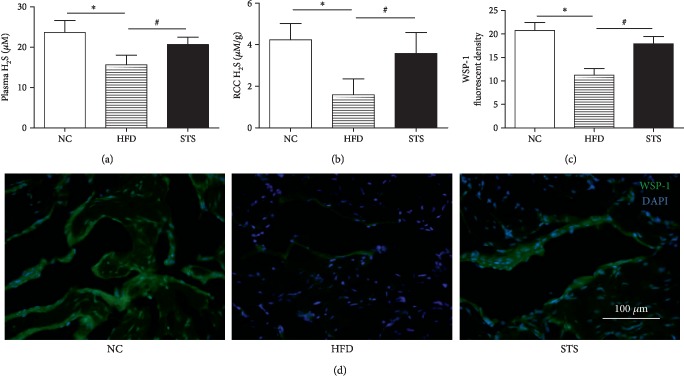
*In vivo* H_2_S production. (a) *In vivo* H_2_S levels in blood. (b) *In vivo* H_2_S levels in penile tissue from each group. (c, d) H_2_S levels were assessed and visualized by a fluorescent H_2_S probe WSP-1 under a fluorescent microscope. Each bar represents mean ± SEM; *N* = 12 rats per group. ^∗^*P* < 0.05 compared to the NC group; ^#^*P* < 0.05 compared with the HFD group.

As can be seen from Figure 3, a significant decrease in the blood and penile levels of H_2_S was detected in the HFD rats, compared to that in NC rats (*P* < 0.05, Figures 3(a) and 3(b)). The STS treatment markedly elevated the H_2_S level in the plasma and penile tissue. (*P* < 0.05, Figures 3(a) and 3(b)) Endogenous H_2_S generation in the penile tissue was measured by specific fluorescent probe WSP-1. Compared to the control, the decreased fluorescent density was observed in corpus cavernosum tissue of HFD rats. WSP-1 fluorescent density increased significantly after STS treatment, which suggested the elevation of endogenous H_2_S generation in the corpus cavernosum induced by STS treatment (*P* < 0.05, Figure 3(c)).

### 3.4. Expression of H_2_S Synthetic Enzymes in HFD Rats

**Figure 4:**
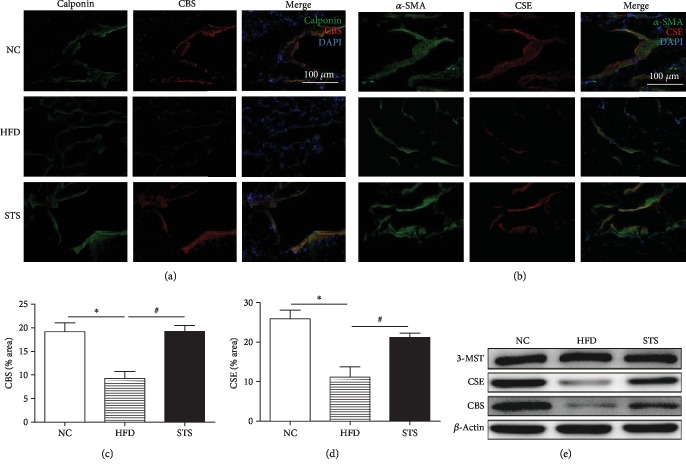
Expression of CBS and CSE in the penile cavernous tissue. (a, b) Immunofluorescence staining for CBS and CSE in penile corpus cavernous tissue in each group. (c, d) Comparison of CBS and CSE expression in different groups. (e) Western blot analysis for CSE, CBS, and 3-MST. Each bar represents mean ± SEM; *N* = 12 rats per group. ^∗^*P* < 0.05 compared to the NC group; ^#^*P* < 0.05 compared with the HFD group.

As evidenced by the significant decrease in expression of CSE and CBS in the penile tissue of the HFD rats as compared to NC rats, hyperlipidemia inhibited the expression of these two H_2_S synthetic enzymes (*P* < 0.05, Figures 4(a) and 4(b)). After STS treatment, the STS group showed significant regeneration of CSE and CBS (*P* < 0.05, Figures 4(a) and 4(b)). The results were also confirmed by Western blot (*P* < 0.05, Figure 4(c)). Double immunofluorescence staining revealed colocalization of H_2_S synthetic enzymes (CSE and CBS) and smooth muscle markers (*α*-SMA and calponin). As shown in Figure 4(c), neither hyperlipidemia nor STS treatment had significant effect on the expression of 3-MST in the penile tissue.

### 3.5. Antioxidative Capability in Serum and Penile Tissue

**Figure 5:**
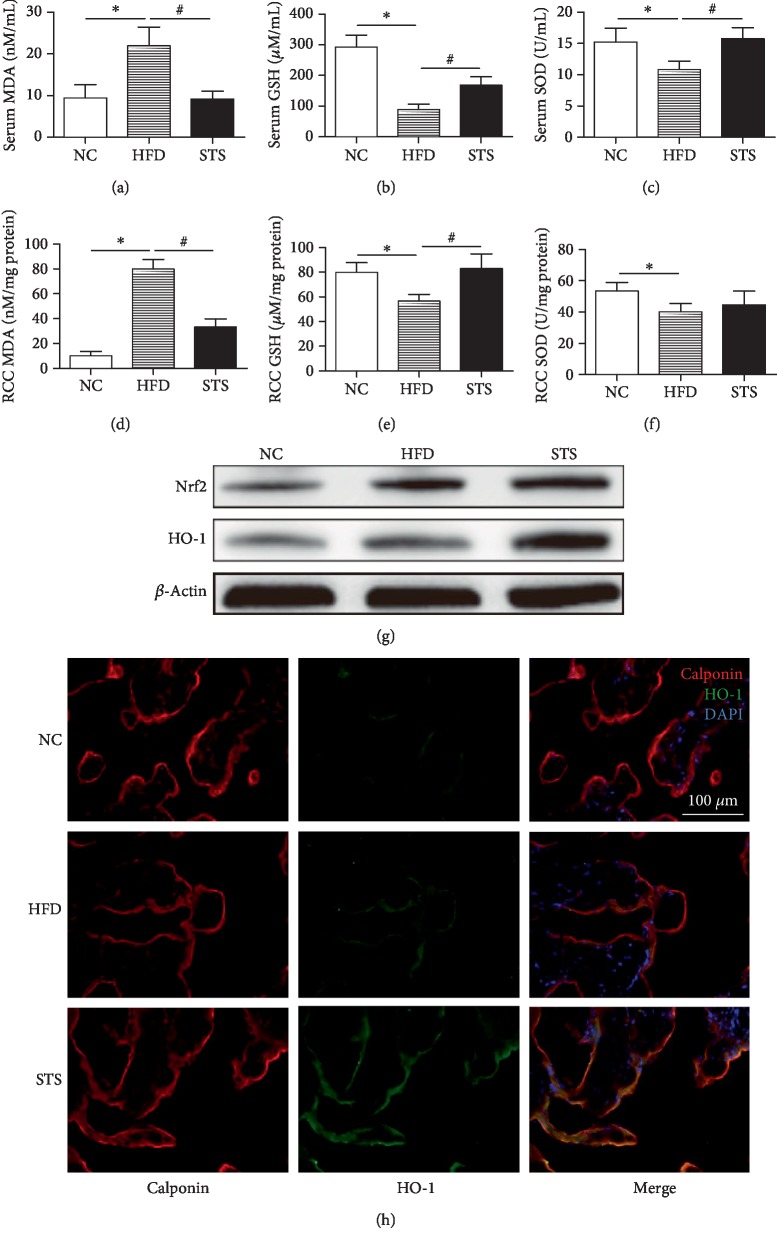
MDA content, antioxidant enzyme activities, and expression of Nrf2/HO-1. (a–c) MDA, GSH, and SOD levels in serum. (d–f) MDA, GSH, and SOD levels in penile corpus cavernous tissue. (g) Western blot analysis for Nrf2 and HO-1 in penile corpus cavernous tissue. (h) Immunofluorescence staining for HO-1 in the penile corpus cavernous tissue. Each bar represents mean ± SEM; *N* = 12 rats per group. ^∗^*P* < 0.05 compared to the NC group; ^#^*P* < 0.05 compared with the HFD group.

As depicted in Figures 5(a)–5(c), there is a significant elevation in MDA and decline in GSH and SOD levels in serum of HFD rats compared with control rats. Treatment with STS remarkably returned this alteration to normalcy (*P* < 0.05, Figures 5(a)–5(c)). Similarly, the STS treatment also significantly elevated the GSH level and reduced MDA contents in the penile tissue (*P* < 0.05, Figures 5(d)–5(f)). As shown in Figure 5(d), the expression rate of antioxidants Nrf2 and HO-1 in the penile tissue of HFD rats was higher compared to that of NC rats. These antioxidants were further expressed with STS treatment (*P* < 0.05, Figure 5(g)). Double immunofluorescence staining showed that HO-1 was mainly expressed in the smooth muscle tissue, which was marked by calponin antibody. The density of HO-1-positive staining in the STS group was significantly higher than that in the HFD group (*P* < 0.05, Figure 5(h)).

### 3.6. H_2_O_2_-Induced Oxidative Stress via the Nrf2/HO-1 Pathway in CSMC

**Figure 6:**
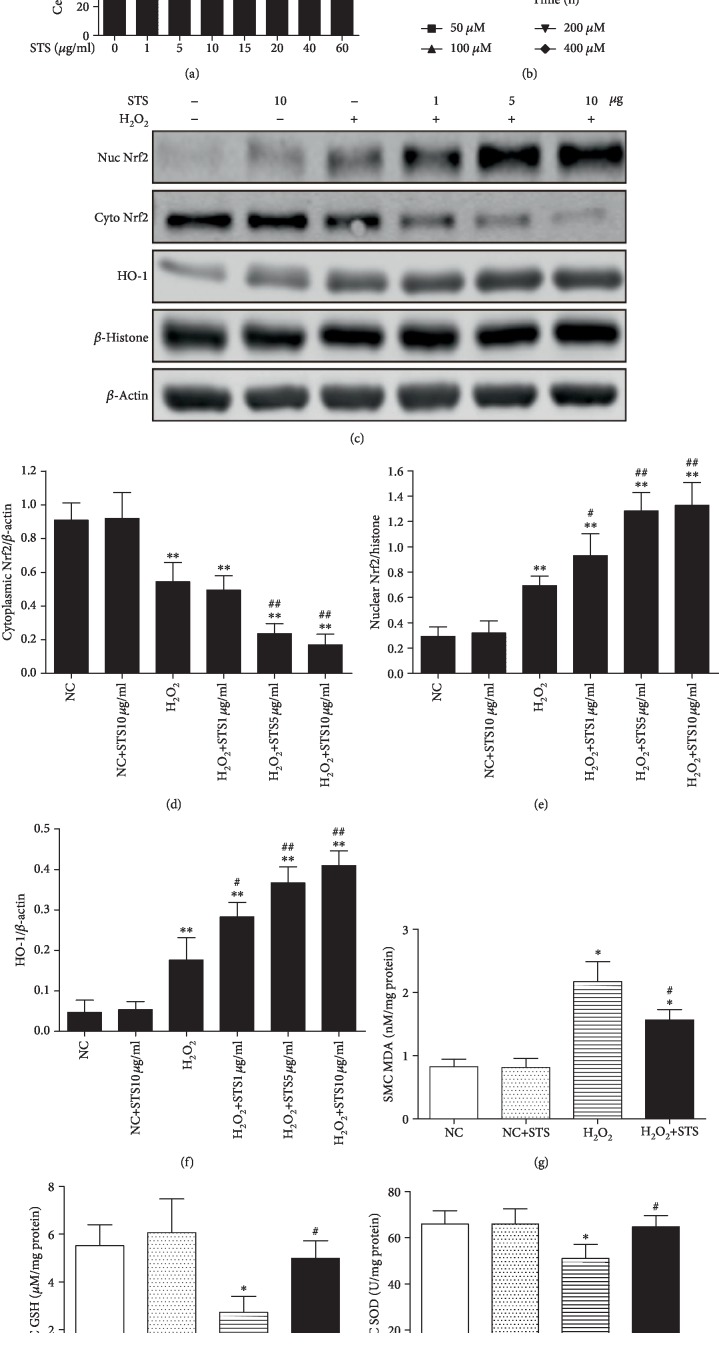
Activation of the Nrf2/HO-1 pathway and antioxidant capacity in STS-pretreated CSMC. (a) Cell viability of STS-treated CSMC. Cells were treated with STS (0-60 *μ*g/ml) for 48 hours, and cell viability was measured by a CCK8 assay. (b) Cell viability of H_2_O_2_-treated CSMC. Cells were treated with H_2_O_2_ (50 *μ*M, 100 *μ*M, 200 *μ*M, and 400 *μ*M) for 6, 12, and 18 hours, and cell viability was measured. (c) Effect of STS pretreatment on the Nrf2/HO-1 pathway in H_2_O_2_-treated CSMC. The CSMC were treated with 100 *μ*M H_2_O_2_ for 12 hours with or without 2 hours of STS (1 *μ*g/ml, 5 *μ*g/ml, and 10 *μ*g/ml) pretreatment, and the expression of Nrf2 and HO-1 was determined by Western blot. (d, e) Effect of STS pretreatment on Nrf2 translocation in H_2_O_2_-treated CSMC. (f) Expression of HO-1 was measured by Western blot. (g, i) Effect of STS pretreatment on antioxidant capacity in H_2_O_2_-treated CSMC. The CSMC were treated with 100 *μ*M H_2_O_2_ for 12 hours with or without 2 hours of 10 *μ*g/ml STS pretreatment, and the MDA, GSH, and SOD levels were measured. Each bar represents mean ± SEM of three independent experiments; *N* = 3 per group. ^∗^*P* < 0.05 compared to the NC group; ^∗∗^*P* < 0.01 compared to the NC group; ^#^*P* < 0.05 compared with the H_2_O_2_ group; ^##^*P* < 0.01 compared with the H_2_O_2_ group.

As demonstrated by the MTT assay, no significant change in CSMC viability was observed after treatment with lower concentrations of STS (0-10 *μ*g/ml) even for 48 hours (*P* > 0.05, Figure 6(a)). In addition, we investigated CSMC death after treatment with different concentrations of H_2_O_2_ and found that H_2_O_2_ induced cell death in a dose-dependent and time-dependent manner (Figure 6(b)). As shown in Figure 6(c), H_2_O_2_ caused an increase of Nrf2 nuclei content and decrease of Nrf2 cytoplasmic content compared to the control group (*P* < 0.05, Figure 6(c)). Pretreatment of STS promoted Nrf2 translocation from the cytoplasm to the nucleus dose dependently, reaching a maximum translocation at 10 *μ*g/ml in H_2_O_2_-treated CSMC (*P* < 0.05, Figures 6(d) and 6(e)). Also, Western blot showed that the increase in HO-1 expression in CSMC exposed to H_2_O_2_ with different doses of STS pretreatment coincided with increased Nrf2 nuclear protein levels (*P* < 0.05, Figure 6(f)). The levels of MDA were markedly increased, and the content of SOD and GSH was decreased in H_2_O_2_-treated cells compared with the control (*P* < 0.05, Figures 6(g)–6(i)). Pretreatment with STS significantly reversed the effect of H_2_O_2_ on the oxidative status of CSMC (MDA, SOD, and GSH) (*P* < 0.05, Figures 6(g)–6(i)). Moreover, all results of the NC+STS group suggested that STS treatment did not significantly affect the indices of antioxidant enzymes under normal conditions (*P* > 0.05, Figures 6(g)–6(i)).

### 3.7. H_2_S Level and Expression of H_2_S-Producing Enzymes in H_2_O_2_-Treated CSMC

**Figure 7:**
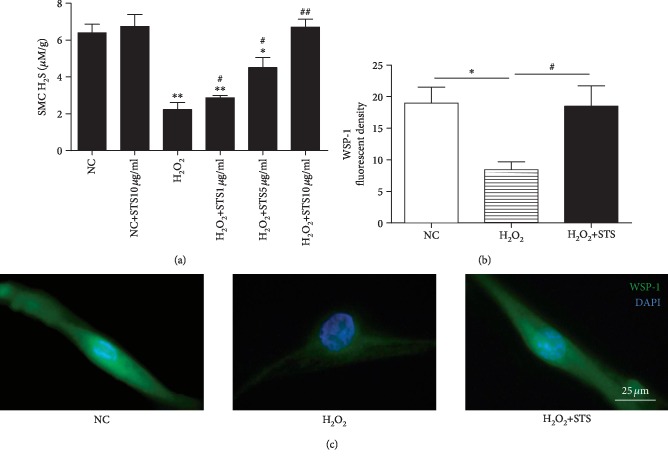
H_2_S production in STS-pretreated CSMC. (a) Effect of STS pretreatment on H_2_S production in H_2_O_2_-treated CSMC. The CSMC were treated with 100 *μ*M H_2_O_2_ for 12 hours with or without 2 hours of STS (1 *μ*g/ml, 5 *μ*g/ml, and 10 *μ*g/ml) pretreatment. The H_2_S production was determined by methylene blue method. (b, c) Cellular H_2_S levels were assessed and visualized by a fluorescent H_2_S probe WSP-1. The CSMC were treated with 100 *μ*M H_2_O_2_ for 12 hours with or without 2 hours of 10 *μ*g/ml STS pretreatment, and WSP-1 florescent density was measured. Each bar represents mean ± SEM of three independent experiments; *N* = 3 per group. ^∗^*P* < 0.05 compared to the NC group; ^∗∗^*P* < 0.01 compared to the NC group; ^#^*P* < 0.05 compared with the H_2_O_2_ group; ^##^*P* < 0.01 compared with the H_2_O_2_ group.

**Figure 8:**
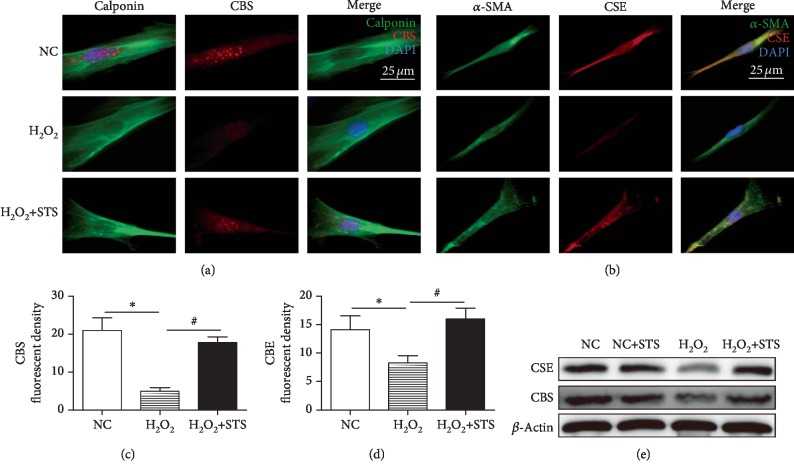
Expression of CBS and CSE in STS-pretreated CSMC. (a–d) Immunofluorescence staining for CBS and CSE in CSMC. The CSMC were treated with 100 *μ*M H_2_O_2_ for 12 hours with or without 2 hours of 10 *μ*g/ml STS pretreatment. Effects of STS pretreatment on expression of CBS and CSE in H_2_O_2_-treated CSMC were detected by immunofluorescence staining. (e) Expression of CSE and CBS was measured by Western blot. Each bar represents mean ± SEM of three independent experiments; *N* = 3 per group. ^∗^*P* < 0.05 compared to the NC group; ^#^*P* < 0.05 compared with the H_2_O_2_ group.

As shown in Figure 7(a), the H_2_S level was significantly decreased following H_2_O_2_ treatment as compared to control (*P* < 0.01, Figure 7(a)). STS showed the tendency to dose dependently increase the H_2_S level in H_2_O_2_-treated CSMC (*P* < 0.05, Figure 7(a)). To further detect the intracellular H_2_S production, WSP-1 was used. Compared to the control, the decreased WSP-1 fluorescent density was observed in H_2_O_2_-treated CSMC (*P* < 0.05, Figures 7(b) and 7(c)). STS pretreatment was found to enhance the intracellular H_2_S content in H_2_O_2_-treated CSMC (*P* < 0.05, Figures 7(b) and 7(c)). As determined using immunofluorescence staining, expression of H_2_S-producing enzymes (CSE and CBS) in the penile tissue was significant lower in the H_2_O_2_-treated group when compared to the control group (*P* < 0.05, Figures 8(a)–8(d)). STS-treated group showed markedly more H_2_S-producing enzyme recovery than that of H_2_O_2_-treated group (*P* < 0.05, Figures 7(a)–7(d)). This was also confirmed by Western blot (*P* < 0.05, Figure 8(e)).

## 4. Discussion

In this study, we demonstrated that *in vivo* and in vitro STS treatment elicited a protective effect against the increased oxidative stress seen in H_2_O_2_-treated CSMC and in penile tissues of HFD-fed rat with hyperlipidemia and, furthermore, that the activation of Nrf2/HO-1 pathway is involved in this protective process. In addition, we provide the first molecular evidence that hyperlipidemia and H_2_O_2_ induces oxidative stress, and this occurs alongside a reduction of H_2_S production and CSE and CBS expression *in vivo* or *in vitro*, which was attenuated by STS treatment, suggesting an antioxidative role of STS. Thus, this study provides significant insights into how STS ameliorated ED in hyperlipidemia rat model and the role of oxidative stress on H_2_S generation *in vivo* and *in vitro*.

HFD-fed rat model has been widely adopted as an experimental method to induce the hyperlipidemia model [17]. The HFD feeding caused a significant increase in body weight and blood fat over the 16-week feeding period. Our findings suggested that administration of STS is able to improve blood lipid profiles (TC, TG, and HDL-C) in the HFD-fed rats, although there is no difference in the body weight. In addition, the activities of antioxidant enzymes (SOD and GSH) and lipid contents (MDA) in the penis and blood were measured to investigate the antioxidant effect of STS on penile tissue damage in the HFD-induced hyperlipidemia rat model. After 16 weeks of the high-fat diet, serum levels of MDA went higher, accompanied with the levels of SOD and GSH going down. The increased MDA and decreased antioxidant enzyme levels (SOD and GSH) confirmed the occurrence of oxidative damage in the penile tissue of the HFD group. This alteration was also observed in the HFD-fed rat model in other study [19]. However, remarkable increases in the SOD and GSH and noteworthy reduction in MDA were observed after treatment with STS. Based on these experimental results, it is demonstrated that STS reduced oxidative stress in the penis caused by high-fat diet, which may explain the recovery of erectile function.

Nrf2 is a key modulator of redox balance and signaling, which drives the expression of a battery of genes that protect against oxidative stress, including GSH, SOD, and HO-1 [20]. HO-1, the best known downstream effector of Nrf2, is regarded as one of the most important intracellular antioxidant mechanisms [11]. Activation of the Nrf2/HO-1 antioxidant pathway contributes to protection from a variety of human diseases [11, 20, 21]. The present study found that hyperlipidemia increases the protein levels of Nrf2 and HO-1 in the penile tissue, which is reflexively activated when suffering from external oxidative stress. In addition, STS could significantly increase the expression of Nrf2 and HO-1, which suggested STS enhanced resistance to oxidative stress in HFD rats. Similar to our results, accumulating evidence has demonstrated that activation of the Nrf2/HO-1 pathway can play a pivotal role in protecting the penis against oxidative stress [22].

Under basal conditions, the activity of Nrf2 is inhibited by Keap1, and upon exposure of cells to oxidative stress, Nrf2 translocates from the cytoplasm to the nucleus and transactivates phase II detoxifying and antioxidant genes [20]. The Nrf2 pathway is vital for the antioxidative response. Therefore, we examined whether STS promotes Nrf2 nuclear translocation, thereby inducing antioxidant gene expression. In the present study, H_2_O_2_ evoked a rapid and transient increase in Nrf2 activation. The increase of nucleus translocation of Nrf2 in H_2_O_2_-treated CSMC underlines the formation of oxidative stress induced by H_2_O_2_. In STS-treated CSMC, STS slightly stimulated Nrf2 translocation. In H_2_O_2_-treated CSMC, the pretreatment of STS promoted further translocation from the cytoplasm to the nucleus dose dependently. As expected, STS pretreatment significantly reduced the ROS production (MDA) and increased the expression of phase II antioxidant genes (HO-1, GSH, and SOD). Thus, STS could inhibit oxidative stress through the Nrf2/HO-1 pathway. These results are consistent with the findings of other study [11]. Since one of the known etiologies of ED is increased ROS formation in the cavernosum, STS could exert protective effect by inhibition of oxidative stress in hyperlipidemia-associated ED [23].

Studies using different species of animals' and humans' corpus cavernosum show evidence of H_2_S's proerectile effects [5, 7, 8]. Our results showed that plasma H_2_S levels were reduced in HFD rats, similar to a previous study [9]. However, H_2_S production and H_2_S-generating enzymes in the corpus cavernosum of hyperlipidemia animal model are rarely reported. We demonstrated that H_2_S production in penile tissues of rats with hyperlipidemia-associated ED was decreased, and STS treatment could alleviate the change. Similar to our findings, decreased H_2_S generation in penile tissues was accompanied by impaired erectile function, indicating the importance of H_2_S in maintaining sexual function [24, 25]. Our results also indicated that the expression of CSE and CBS was decreased in the HFD group, and STS treatment increased expression of these two H_2_S-generating enzymes, which can account for the H_2_S production changes. Interestingly, immunofluorescent staining showed that CBS and CSE which colocalize with *α*-SMA and calponin indicated that H_2_S-generating enzymes were mainly localized in the smooth muscle tissue in the cavernosum. Researchers also found similar results in human penile tissue [5]. Western blot experiments also showed the expression of 3-MST in the penile tissue, consistent with previous studies [24, 26]. Nevertheless, 3-MST expression did not change in penile tissues from rats with hyperlipidemia, indicating the downregulation of CSE and CBS might contribute to the decreased H_2_S production in the rats with hyperlipidemia. Since hyperlipidemia results in oxidative stress, to further clarify the role of oxidative stress in H_2_S formation, the expression of CSE and CBS and H_2_S levels in CSMC under oxidative stress were determined. Our data suggested that oxidative stress inhibited the expression of CSE and CBS, causing decreased H_2_S production. Similar to our findings, a study in fat-fed rat shows that vascular CSE expression is reduced during oxidative stress [9]. Further studies are warranted to identify the exact mechanism of this phenomenon.

## 5. Conclusion

Our data suggest that hyperlipidemia and the related oxidative stress significantly reduced H_2_S production and CSE and CBS expression in vivo and in vitro. Treatment with STS may preserve erectile function by elevating the H_2_S formation and H_2_S-generating enzyme expression and enhancing antioxidant capacity via the Nrf2/HO-1 pathway in rats with hyperlipidemia.

## Figures and Tables

**Table 1:** Antibodies used in this study.

	*Primary antibody*	*Species/types*	*Dilution*	*Catalogue number*	*Product information*
H_2_S-generating enzyme markers	CSE	Mouse mAb	IF 1 : 50 WB 1 : 2000	60234-1-Ig	Proteintech, Rosemont, IL, USA
	CBS	Rabbit pAb	IF 1 : 50 WB 1 : 2000	14787-1-AP	Proteintech, Rosemont, IL, USA
	3-MST	Rabbit pAb	WB 1 : 500	NBP1-82617	Novus Biologicals, Centennial, CO, USA
Antioxidant enzyme markers	Nrf2	Rabbit pAb	WB 1 : 1000	ab137550	Abcam, Cambridge, MA, USA
	HO-1	Rabbit pAb	IF 1 : 100 WB 1 : 1500	27282-1-AP	Proteintech, Rosemont, IL, USA
Smooth muscle markers	*α*-SMA	Rabbit pAb	IF 1 : 50	14395-1-AP	Proteintech, Rosemont, IL, USA
	Calponin	Mouse mAb	IF 1 : 50	sc-58707	Santa Cruz Biotechnology, Santa Cruz, CA, USA

	*Secondary antibody*	*Types*	*Dilution*	*Catalogue number*	*Product information*
FITC conjugate	Goat anti-mouse	IgG (H+L)	IF 1 : 50	SA00003-1	Proteintech, Rosemont, IL, USA
	Goat anti-rabbit	IgG (H+L)	IF 1 : 50	SA00003-2	Proteintech, Rosemont, IL, USA
Cy3 conjugate	Goat anti-mouse	IgG (H+L)	IF 1 : 50	SA00009-1	Proteintech, Rosemont, IL, USA
	Goat anti-rabbit	IgG (H+L)	IF 1 : 50	SA00009-2	Proteintech, Rosemont, IL, USA

IF: immunofluorescence; mAb: monoclonal antibody; pAb: polyclonal antibody; WB: Western blot.

## Data Availability

The data that support the findings of this study are available on request from the corresponding author, Dr. Wei (email: profwei@126.com). The data are not publicly available because the research containing information that could compromise the privacy of research participants.
