# Corrigendum to “Sodium Tanshinone IIA Sulfonate Attenuates Erectile Dysfunction in Rats with Hyperlipidemia”

**DOI:** 10.1155/2020/1062074

**Published:** 2020-10-15

**Authors:** Liren Zhong, Wei Ding, Qinyu Zeng, Binglin He, Haibo Zhang, Li Wang, Junhong Fan, Shuhua He, Yuanyuan Zhang, Anyang Wei

**Affiliations:** ^1^Department of Urology, Nanfang Hospital, Southern Medical University, Guangzhou, Guangdong, China; ^2^Department of Urology, The First Affiliated Hospital of Guizhou University of Traditional Chinese Medicine, Guiyang, Guizhou, China; ^3^Wake Forest Institute of Regenerative Medicine University, Wake Forest School of Medicine, Winston-Salem, North Carolina, USA

In the article titled “Sodium Tanshinone IIA Sulfonate Attenuates Erectile Dysfunction in Rats with Hyperlipidemia” [1], there was a spelling error in author Qinyu Zeng's name in the author list, where “Qingyu Zeng” should have read “Qinyu Zeng.” This is corrected as shown above.
